# Temporal profiling of plasma cytokines, chemokines and growth factors from mild, severe and fatal COVID-19 patients

**DOI:** 10.1038/s41392-020-0211-1

**Published:** 2020-06-19

**Authors:** Zhi-Sheng Xu, Ting Shu, Liang Kang, Di Wu, Xing Zhou, Bo-Wei Liao, Xiu-Lian Sun, Xi Zhou, Yan-Yi Wang

**Affiliations:** 1 Key Laboratory of Special Pathogens and Biosafety, Wuhan Institute of Virology, Center for Biosafety Mega-Science, Chinese Academy of Sciences, Wuhan, 430071 China; 2 The Joint Center of Translational Precision Medicine, Guangzhou Institute of Pediatrics-Wuhan Institute of Virology, Guangzhou Women and Children’s Medical Center, Guangzhou, 510623 China; 3 The Joint Laboratory of Infectious Diseases and Health, Wuhan Institute of Virology & Wuhan Jinyintan Hospital, Wuhan Institute of Virology, Chinese Academy of Sciences, Wuhan, 430071 China; 4 The Joint Laboratory of Infectious Diseases and Health, Wuhan Institute of Virology & Wuhan Jinyintan Hospital, Wuhan Jinyintan Hospital, Wuhan, 430023 China

**Keywords:** Infectious diseases, Immunological disorders

**Dear Editor,**

In December 2019, a novel coronavirus that is related to severe acute respiratory syndrome coronavirus (SARS-CoV) and Middle East respiratory syndrome coronavirus (MERS-CoV) in phylogenetic distance was identified.^1^ This virus, which was later designated as SARS-CoV-2, also causes acute respiratory disease syndrome (ARDS) termed coronavirus disease 2019 (COVID-19), which was declared as a pandemic by the World Health Organization in March 2020.

Although the majority of patients of COVID-19 show moderate symptoms such as dry cough and fatigue, up to 20% cases develop severe symptoms characterized as ARDS, a clinical pulmonary phenomenon marked by the development of bilateral infiltrates and hypoxemia.^2^ The median incubation period of SARS-CoV-2 infection is ~4–5 days before symptom onset, and the majority of symptomatic patients develop symptoms within 11.5 days. Within 5–6 days of symptom onset, SARS-CoV-2 viral load reaches its peak, which is much earlier than SARS-CoV.^3^ Severe COVID-19 cases progress to ARDS with hypoxemia around 8–12 days after symptom onset.^2^ Although various independent factors such as older age and existing diseases contribute to mortality, the majority of fatal patients die of complications such as ARDS, myocardial injury, acute kidney injury, and sepsis.^2,3^

The pathogenesis of COVID-19 has been heavily investigated in the past months. Pathophysiology in COVID-19 is characterized by diffuse alveolar damage, focal reactive hyperplasia of pneumocytes, inflammatory cellular infiltration, vasculitis, hypercoagulability, neutrophilia, and lymphopenia.^4^ Studies have suggested that hyper-inflammation is linked to more severe disease of COVID-19, which is characterized by a cytokine releasing syndrome (CRS).^3,5^ It has been reported that some inflammatory cytokines (such as IL-6, IL-10, and TNF-α) and chemokines (such as CXCL10/ IP-10, CCL2/MCP-1, and CCL3/MIP-1α) are upregulated in COVID-19 patients.^2^ However, these studies are limited by the small sample size, narrowed cytokine and chemokine spectrum, and absence of temporal kinetic analysis of these factors with disease progression. Currently, limited information is available on host factors and biomarkers affecting individual outcomes in COVID-19. Identification of host plasma factors that are correlated to COVID-19 progression may provide potential biomarkers and targets for developing therapeutics.

To systematically investigate the kinetic changes of plasma levels of cytokines, chemokines and growth factors (CCGFs) over the disease courses in COVID-19 patients as well as the correlations between the CCGF profiles and disease severity, we measured levels of 48 CCGFs in plasma of mild, severe and fatal COVID-19 patients collected at different stages of disease courses. We collected sera from 6 fatal, 7 severe, and 10 mild patients at day 1, 5, 10, and 14 after diagnosis. One sample was collected from each of the 4 healthy donors. We measured the levels of CCGFs in these samples utilizing a multiplex system for simultaneous detection of 48 CCGFs. As shown in Supplementary Fig. S1, the levels of 7 CCGFs, including IL-3, IL-10, IL-12 p70, IL-15, IL-17A, β-NGF, and GM-CSF, were comparable between COVID-19 patients and healthy individuals, and were not markedly changed in all mild, severe and fatal patients throughout the disease courses. The other 41 CCGFs were significantly elevated in COVID patients, and these elevations were differentially correlated to disease severity as described below.

The plasma levels of 20 CCGFs, including IL-1α, IL-1β, IL-4, IL-5, IL-7, IL-12 p40, IL-13, IL-16, TNF-α, TRAIL, IFN-α2, CXCL1/GRO-α, CXCL12/SDF-1α, CCL11/Eotaxin, CCL27/CTACK, G-CSF, LIF, MIF, SCGF, and VEGF were elevated in all three groups of patients (Fig. 1a and Supplementary Fig. S2). The levels of these CCGFs remained relatively steady over the disease periods, and had no significant differences among all three COVID-19 groups of patients. Among them, levels of MIF, SCGF, CXCL1, and CCL27 were elevated to levels of >500 pg/ml; levels of VEGF, IL-12 p40, IL-16, TNF-α, and G-CSF were elevated to the range of 50–500 pg/ml, and the other CCGFs were elevated to <50 pg/ml (Fig. 1a and Supplementary Fig. S2). On the other hand, the levels of CCL11, CCL27, and CXCL12 were more than 10-fold higher in COVID-19 patients comparing to healthy donors. The other 17 CCGFs in this category were induced for less than 10 folds in COVID-19 patients (Supplementary Fig. S3).

**Fig. 1:**
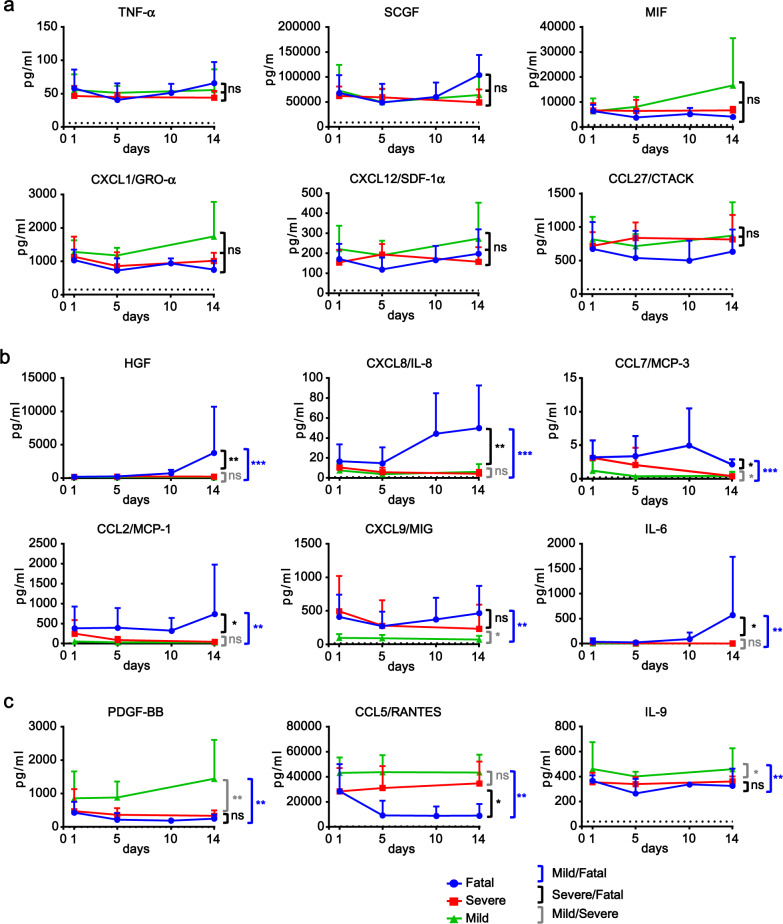
Levels of CCGFs increased in COVID-19 patients. **a** Levels of CCGFs that were increased in COVID-19 patients but not correlated to disease severity. **b** Levels of CCGFs that were increased in COVID-19 patients and positively correlated to COVID-19 disease severity. **c** Levels of CCGFs that were increased in COVID-19 patients and negatively correlated to COVID-19 disease severity. Dynamic levels of the indicated CCGFs in plasma samples of mild (*n* = 10), severe (*n* = 7), and fatal (*n* = 6) COVID-19 patients at the indicated days after diagnosis as well as healthy individuals (n = 4) were measured by Bio-plex. Data shown are averages plus SD of the CCGF levels in mild, severe, and fatal COVID-19 patients, and averages of the CCGF levels in healthy individuals (dotted line). *p < 0.05; **p < 0.01 (Turkey’s test as described in Supplementary Information)

The plasma levels of 16 CCGFs, including HGF, CXCL8/IL-8, CCL7/MCP-3, CCL2/MCP-1, CXCL9/MIG, CXCL10/ IP-10, IL-6, IL-18, IL-2, M-CSF, IL-1Rα, IL-2Rα/CD25, IFN-γ, CCL3/MIP-1α, basic FGF, and SCF were significantly higher in fatal than severe and/or mild COVID-19 patients (Fig. 1b and Supplementary Figs. S4 and S5). Twelve of the CCGFs, including IFN-γ, IL-1Rα, IL-2, IL-2Rα, IL-6, CXCL8, IL18, CCL2, CCL3, SCF, HGF, and basic FGF, were upregulated to similar levels at the early stages (such as 1 and/or 5 days after diagnosis) in all COVID-19 patients. However, these CCGFs were markedly further upregulated in fatal patients at late stages especially day 14 after diagnosis, while remained at steady levels at late stages in mild and severe patients. Therefore, the significant upregulation of these CCGFs at late stages is correlated to the fatality of COVID-19 patients. Four of the CCGFs, including M-CSF, CXCL9, CCL7, and CXCL10, were upregulated to higher levels in fatal than severe and mild patients. However, these CCGFs were not further upregulated or even decreased in late stages of disease progression, suggesting that these CCGFs are correlated to disease severity but not further fatality of the COVID-19 patients.

Levels of 5 CCGFs, including PDGF-BB, CCL5/RANTES, CCL4/MIP-1β, IL-9, and TNF-β were upregulated in all COVID-19 patients but negatively correlated to disease severity (Fig. 1c and Supplementary Fig. S6). These CCGFs were expressed at significantly higher levels in mild than the severe and/or fatal COVID-19 patients. These results suggest that the levels of these CCGFs are correlated to recovery and beneficial effects of COVID-19 patients.

In conclusion, our results suggest that SARS-CoV-2 infection induces an extensive CRS, which contributes to the pathogenesis of COVID-19. The primary proinflammatory cytokines (such as TNF-α, IL-1α, and IL-1β), Th2-type cytokines (IL-4, 5, and 13), certain inflammatory chemokines (CXCL1/GRO-α, CCL11/Eotaxin, CCL27/CTACK and CXCL12/SDF-1α) and growth factors (LIF, VEGF, SCGF, and G-CSF) are upregulated in mild, severe and fatal COVID-19 patients. It is possible that these CCGFs are involved in common pulmonary inflammation and respiratory symptoms in COVID-19 patients. Upregulation of various CCGFs at the late stages of COVID-19, including the Th1-type cytokines (IL-2, IL-18, and IFN-γ), IL-6, the growth factors M-CSF, SCF, HGF, and basic FGF, the chemokines CCL2/MCP-1, CCL3/MIP-1α, CCL7/MCP-3, CXCL8/IL-8, CXCL9/MIG, and CXCL10/IP-10, are correlated to the fatality of COVID-19 patients. It is interesting that many of these CCGFs are involved in promoting inflammation and tissue damage. Five CCGFs (PDGF-BB, CCL5/RANTES, IL-9, TNF-β, and CCL4/MIP-1β) are upregulated to higher levels in mild than severe and/or fatal COVID-19 patients. Some of these CCGFs play important roles in resolution of inflammation and healing of tissue damage. Taken together, our studies suggest that heightened inflammation and tissue damage as well as the impaired resolution of inflammation and healing of tissue damage mediated by various CCGFs contribute to disease severity and particularly fatality of COVID-19 patients. The temporal changes of the identified CCGFs may serve as biomarkers for prognosis of COVID-19 patients. Some of the identified CCGFs may serve as targets for the development of therapeutics. In this context, the recent clinical trial of the IL-6 receptor antibody tocilizumab for the treatment of severe COVID-19 provides an example.^6^

## Supplementary information

Supplementary Information
