# Supplementary material for: Temporal profiling of plasma cytokines, chemokines and growth factors from mild, severe and fatal COVID-19 patients
Source: Signal Transduct Target Ther. 2020 Jun 19;5:100. doi: 10.1038/s41392-020-0211-1 (PMC7303571; doi:10.1038/s41392-020-0211-1)
Supplement: Supplementary file 1 — Supplementary Information [file 41392_2020_211_MOESM1_ESM.pdf]

Supplementary Materials for

Temporal profiling of plasma cytokines, chemokines and growth factors from mild, severe and fatal COVID-19 patients

Zhi-Sheng Xu[1,4#], Ting Shu[2#], Liang Kang[3#], Di Wu[2#], Xing Zhou[3#], Bo-Wei Liao[1], Xiu-Lian Sun[1], Xi Zhou[2], Yan-Yi Wang[1,2]*

Correspondence to: wangyy@wh.iov.cn

This PDF file includes:

Materials and Methods

Figures S1 to S6

Materials and Methods

Ethics and Human Subjects

All work performed in this study was approved by the Wuhan Jinyintan Hospital Ethics Committee and written informed consents were obtained from patients. Diagnosis of SARS-CoV-2 infection, pneumonia and clinical classification was based on the New Coronavirus Pneumonia Diagnosis and Treatment Plan (6th edition) published by the National Health Commission of China. Briefly, laboratory confirmed patients with fever, respiratory manifestations and radiological findings indicative of pneumonia were considered as mild cases, while patients who met any of the following were considered as severe patients: 1) respiratory distraction (respiration rate≥30/min; 2) resting oxygen saturation≤93%, or 3) arterial oxygen partial pressure (PaO_2) / fraction of inspired oxygen (FiO_2) ≤300 mmHg (1 mmHg = 0.133 kPa). Patients who experienced deterioration and finally progressed to death were considered as fatal patients. All patients (age 55.2±14.8) that enrolled in this study were subjected to injection of cefoperagone sodium and tazobactam sodium for anti-bacterial therapy, and 8 patients including 4 fatal patients and 4 severe patients were also subjected to additional injection of methylprednisolone sodium succinate for therapy.

Patient Samples

All patients were recruited by Jinyintan Hospital in Wuhan. Blood samples (≤3mL each) from fatal COVID-19 patients were collected over the course of their diseases (approximately 1, 5, 10, 14 days after diagnosis respectively). These patients died at 18.4±6 days after hospitalization. Blood samples (≤3mL each) from patients with severe and mild symptoms were collected at approximately 1, 5 (the time when the disease was most serious), 14 (the time before discharge) days respectively. Single samples were collected from healthy donors recruited from healthcare workers and laboratory workers at Wuhan Jinyintan Hospital and Wuhan Institute of Virology of the Chinese Academy of Sciences, and none of whom had previously experienced

SARS-CoV-2 infection. All samples were collected using potassium-EDTA blood collection tubes. All the blood samples were treated according to the biocontainment procedures for processing of SARS-CoV-2-positive samples. The blood samples were centrifuged at 1,700 g for 10 min at room temperature to remove cells and the plasma were transferred to screw-cap vials and attached with the labels, and then stored at -80℃.

Multiplex Analysis

The levels of plasma cytokines were analyzed using Bio-Plex (Bio-Rad Laboratories, Inc.) multiplex magnetic bead-based antibody detection kits following the manufacturer's instructions. The Bio-Plex Pro Human Cytokine 48-Plex Screening Panels (Cat #12007283) were used for analysis of 48 protein factors according to the protocols provided by the manufacturer.

Statistical analyses

The cytokine levels were log10 transformed to fit the normal distribution. The main effects of severity and time on the cytokine levels and the interactions between the severity and time were evaluated by repeated measures ANOVA using the 'nlme' package in R. The marginal means of CCGFs' levels over the time points were compared by the Tukey's honestly significant difference test.

Figure S1

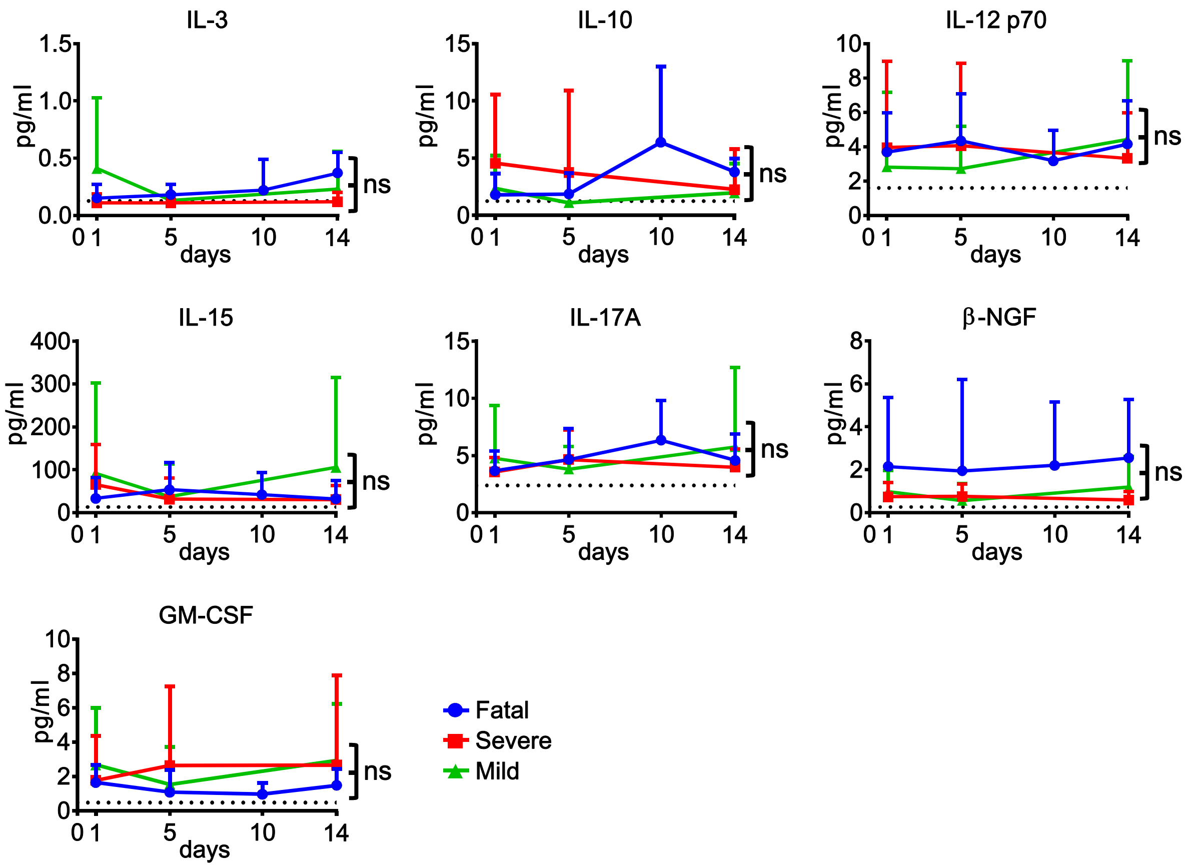

Fig. S1. Levels of CCGFs not markedly changed in COVID-19 patients. Dynamic levels of the indicated CCGFs in plasma samples of mild (n=10), severe (n=7), and fatal (n=6) COVID-19 patients at the indicated days after diagnosis as well as healthy individuals (n=4) were measured by Bio-plex. Data shown are averages plus SD of the CCGF levels in mild, severe and fatal COVID-19 patients, and averages of the CCGF levels in healthy individuals (dotted line). ns, not significant (Turkey's test as described in Supplementary Materials and Methods).

Figure S2

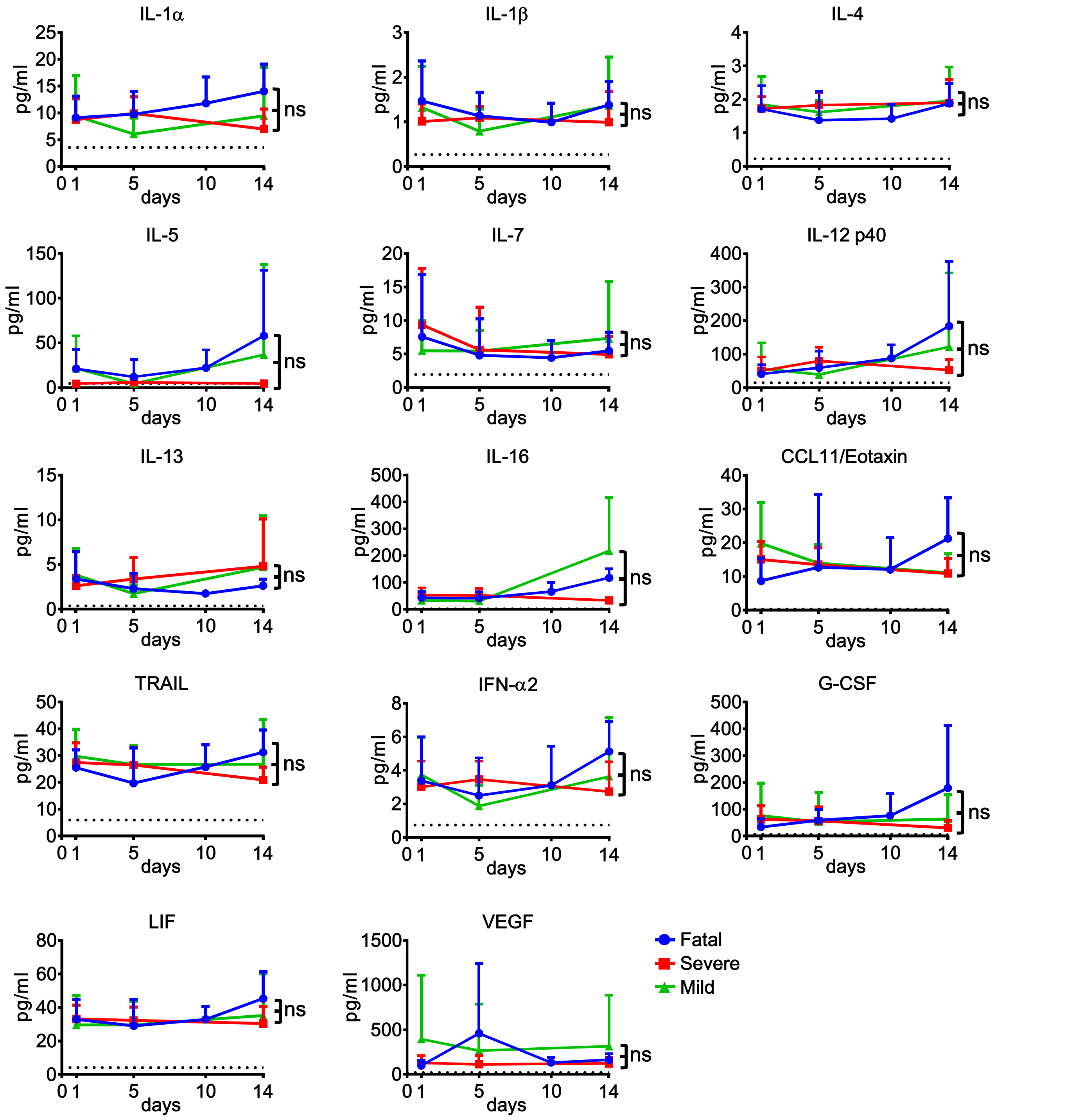

Fig. S2. Levels of CCGFs were increased in COVID-19 patients but not correlated with disease progression. Dynamic levels of the indicated CCGFs in plasma samples of mild (n=10), severe (n=7), and fatal (n=6) COVID-19 patients at the indicated days after diagnosis as well as healthy individuals (n=4) were measured by Bio-plex. Data shown are averages plus SD of the CCGF levels in mild, severe and fatal COVID-19 patients, and averages of the CCGF levels in healthy individuals (dotted line). ns, not siginficant (Turkey's test as described in Supplementary Information).

Figure S3

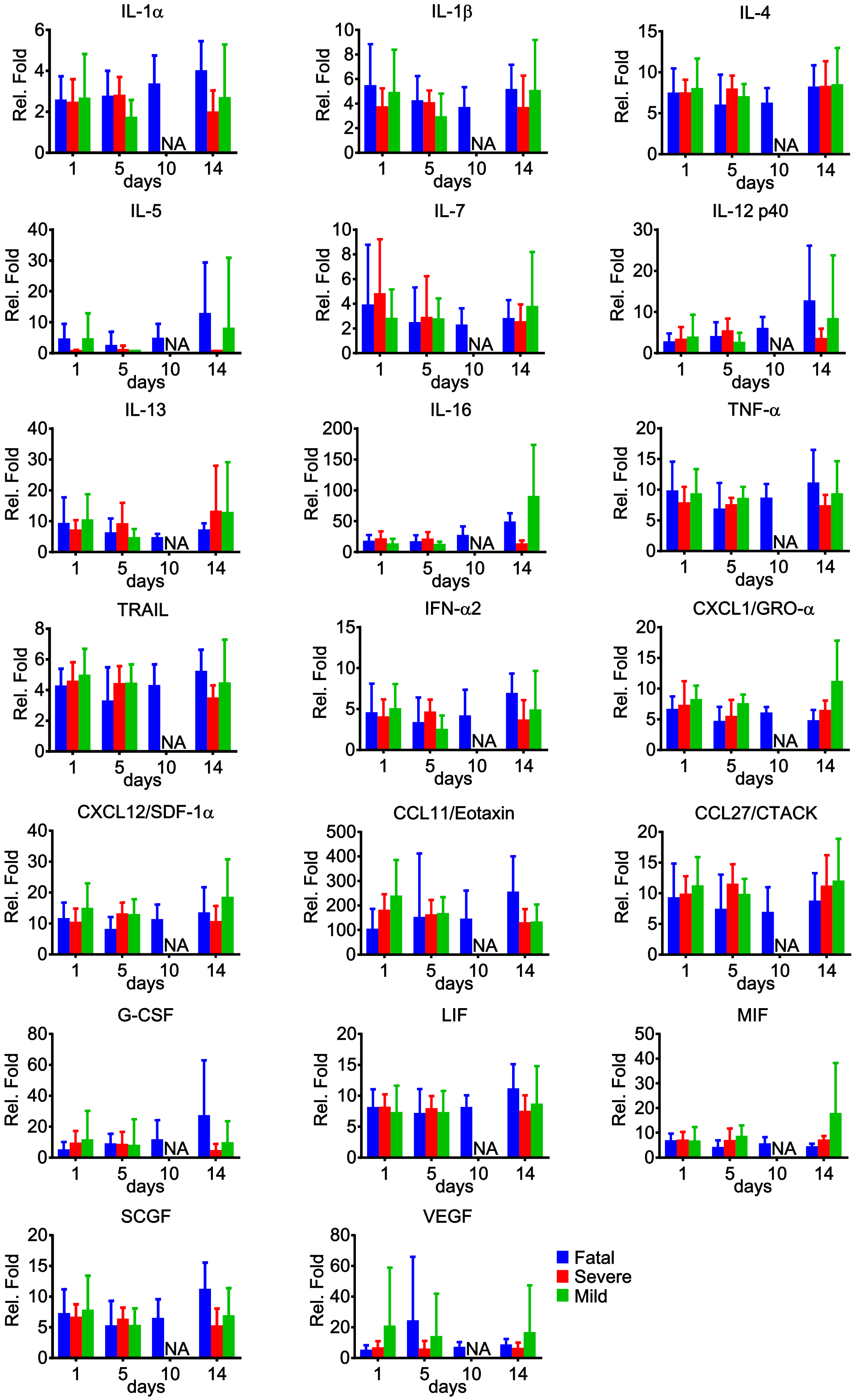

Fig. S3. Fold changes of CCGFs that were increased in COVID-19 patients but not correlated with disease progression. NA, not available.

Figure S4

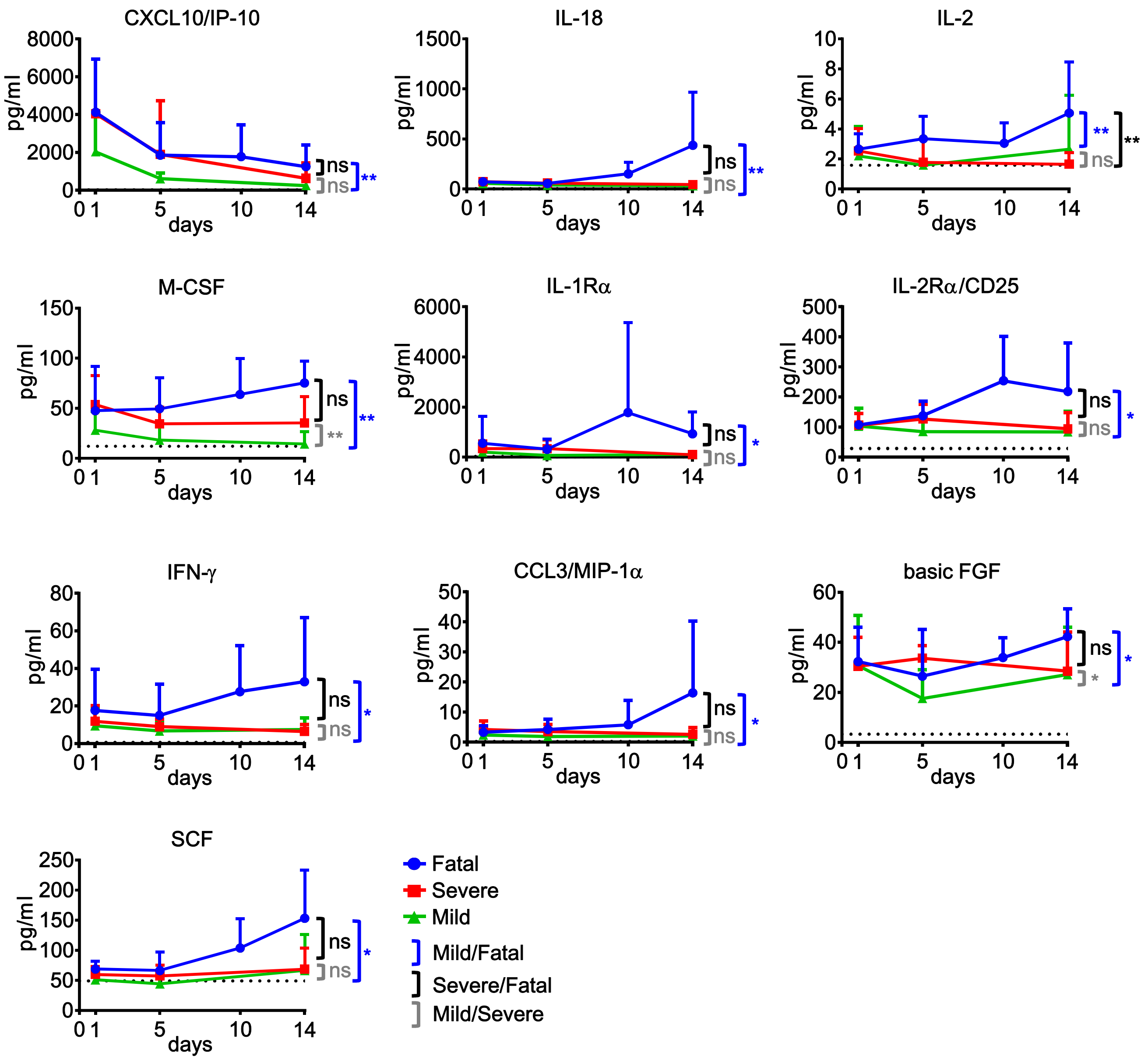

Fig. S4. Levels of CCGFs positively correlated to disease severity. Dynamic levels of the indicated CCGFs in plasma samples of mild (n=10), severe (n=7), and fatal (n=6) COVID-19 patients at the indicated days after diagnosis as well as healthy individuals (n=4) were measured by Bio-plex. Data shown are averages plus SD of the CCGF levels in mild, severe and fatal COVID-19 patients, and averages of the CCGF levels in healthy individuals (dotted line). ns, not significant; *, p<0.05; **, P<0.01 (Turkey's test as described in Supplementary Information).

Figure S5

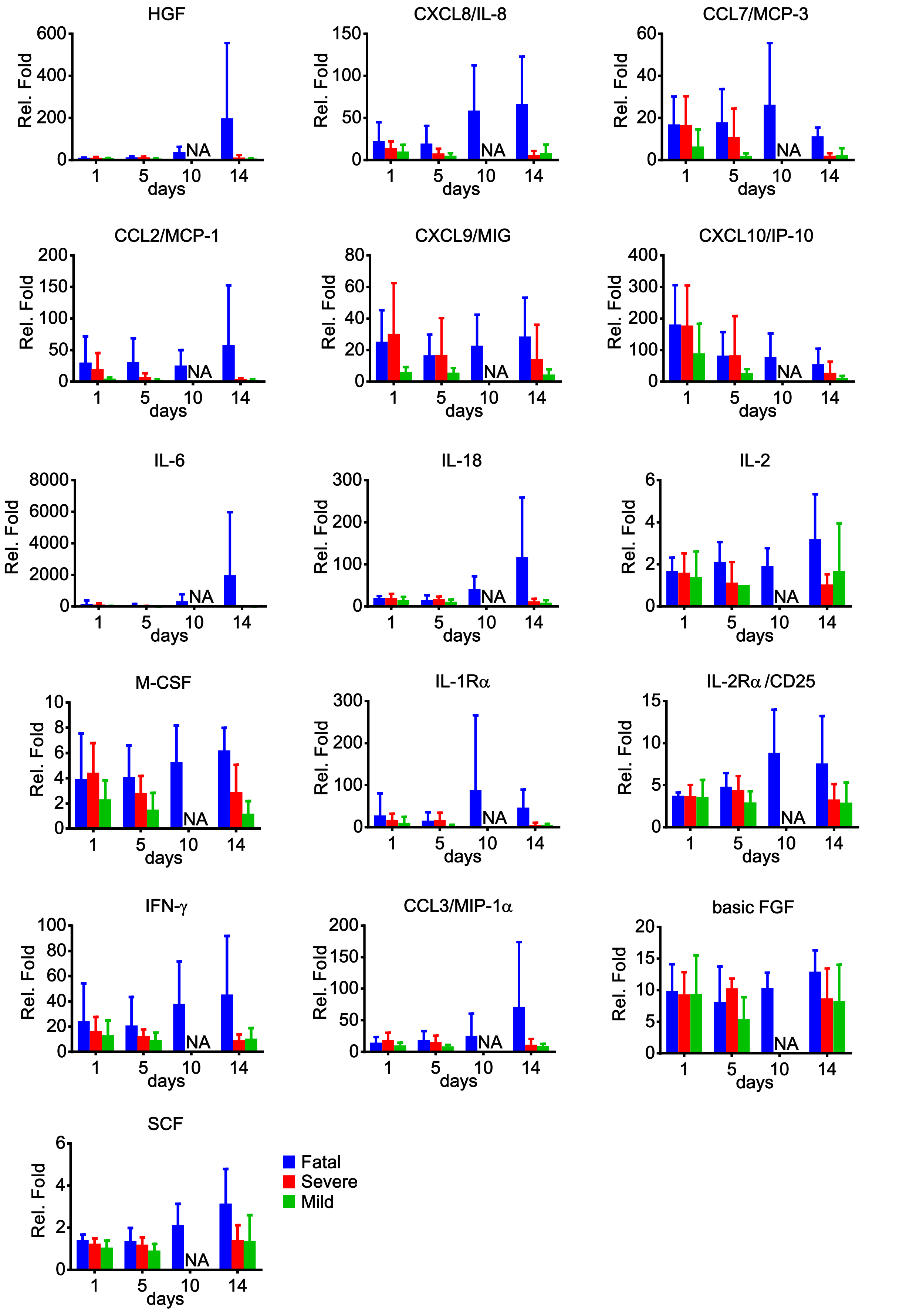

Fig. S5. Fold changes of CCGFs positively correlated to disease severity. NA, not available.

Figure S6

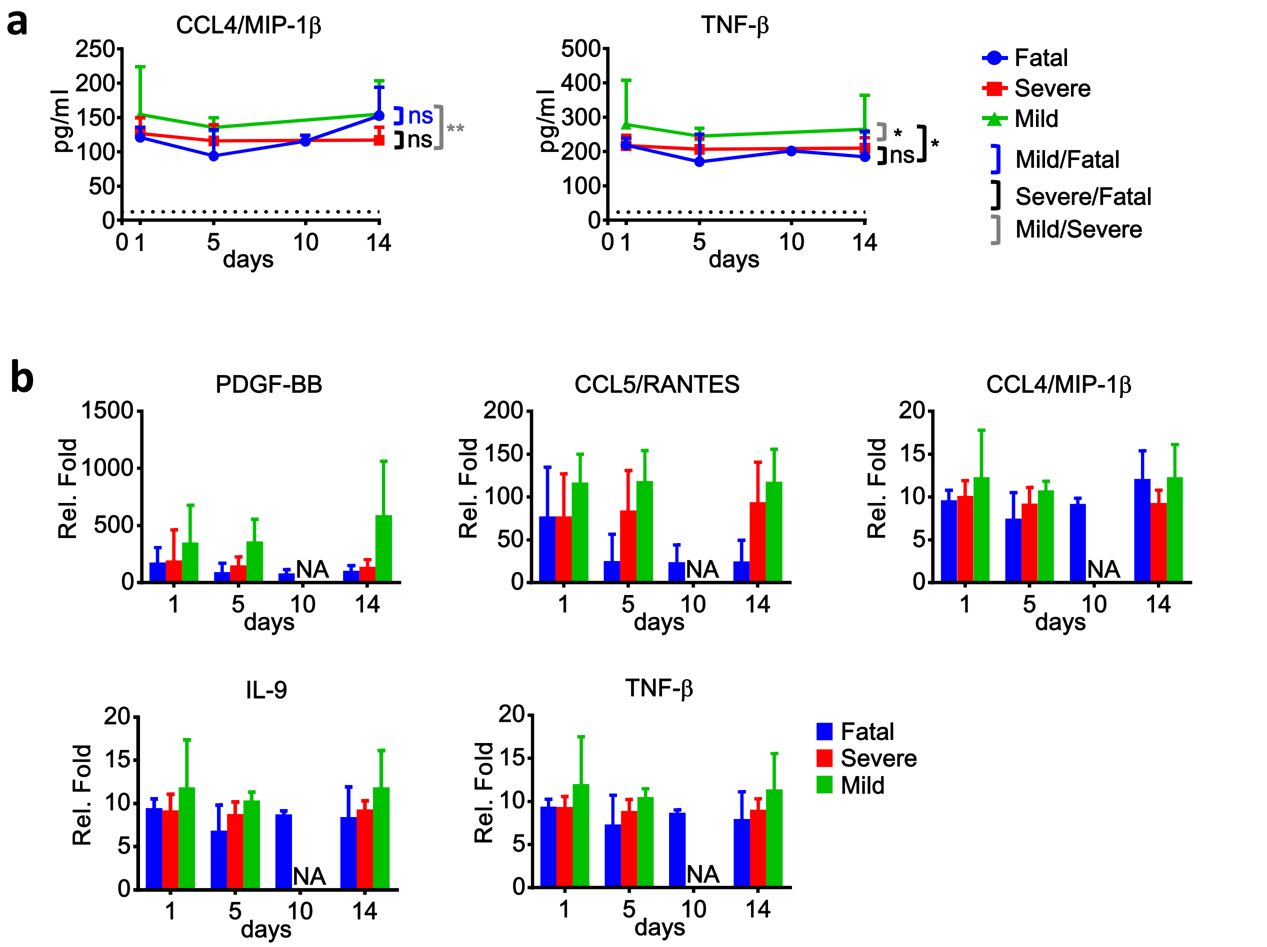

Fig. S6. Levels of CCGFs negatively correlated to disease severity.
a Dynamic levels of the indicated CCGFs in plasma samples of mild (n=10), severe (n=7), and fatal (n=6) COVID-19 patients at the indicated days after diagnosis as well as healthy individuals (n=4) were measured by Bio-plex. Data shown are averages plus SD of the CCGF levels in mild, severe and fatal COVID-19 patients, and averages of the CCGF levels in healthy individuals (dotted line). ns, not significant; *, p<0.05; **, P<0.01 (Turkey's test as described in Supplementary Information).
b Fold changes of CCGFs negatively correlated to disease severity. NA, not available.
